# Next-generation time of death estimation: combining surrogate model-based parameter optimization and numerical thermodynamics

**DOI:** 10.1098/rsos.220162

**Published:** 2022-07-27

**Authors:** Leah S. Wilk, Gerda J. Edelman, Maurice C. G. Aalders

**Affiliations:** ^1^ Department of Biomedical Engineering and Physics, Amsterdam UMC Location AMC, University of Amsterdam, Meibergdreef 9, 1105AZ Amsterdam, The Netherlands; ^2^ Co van Ledden Hulsebosch Center, University of Amsterdam, Science Park 904, 1098XH Amsterdam, The Netherlands; ^3^ Netherlands Forensic Institute, Divisie Bijzondere Dienstverlening en Expertise, Laan van Ypenburg 6, 2497 GB The Hague, The Netherlands

**Keywords:** postmortem interval estimation, forensic science, numerical thermodynamics, surrogate model-based parameter optimization, skin thermometry

## Abstract

The postmortem interval (PMI), i.e. the time since death, plays a key role in forensic investigations, as it aids in the reconstruction of the timeline of events. Currently, the standard method for PMI estimation empirically correlates rectal temperatures and PMIs, frequently necessitating subjective correction factors. To address this shortcoming, numerical thermodynamic algorithms have recently been developed, providing rigorous methods to simulate postmortem body temperatures. Comparing these with measured body temperatures then allows non-subjective PMI determination. This approach, however, hinges on knowledge of two thermodynamic input parameters, which are often irretrievable in forensic practice: the ambient temperature prior to discovery of the body and the body temperature at the time of death (perimortem). Here, we overcome this critical limitation by combining numerical thermodynamic modelling with surrogate model-based parameter optimization. This hybrid computational framework predicts the two unknown parameters directly from the measured postmortem body temperatures. Moreover, by substantially reducing computation times (compared with conventional optimization algorithms), this powerful approach is uniquely suited for use directly at the crime scene. Crucially, we validated this method on deceased human bodies and achieved the lowest PMI estimation errors to date (0.18 h ± 0.77 h). Together, these aspects fundamentally expand the applicability of numerical thermodynamic PMI estimation.

## 1. Introduction

Accurate estimation of the postmortem interval (PMI), i.e. the time since death, is essential in reconstructing an antemortem timeline of events and therefore plays a crucial role in forensic investigations. As a result, a variety of pathophysiological changes, which scale with the time since death, have been studied as potential measures of the PMI [1–10]. The pathophysiological change most commonly studied in this context is the postmortem change in body temperature (*algor mortis*) [11–20], as it is easily and rapidly probed directly at the crime scene, requiring minimal operational resources. Accordingly, the current standard method for early PMI estimation (Henssge's nomogram) empirically correlates the rectal temperature and the PMI [21,22]. Despite its wide application, this approach is subject to several limitations, notably its use of subjective correction factors [23,24]. Recently, more rigorous approaches for thermometric PMI estimation have been developed, which simulate postmortem body cooling by computing the underlying thermal energy transfer within numerical frameworks, such as finite differences [25,26] or finite elements [27,28]. While these are powerful approaches, allowing non-subjective and even non-contact [29] thermometric PMI reconstruction, they necessitate precise knowledge of the case-specific thermodynamic input parameters. In forensic practice, however, some of these input parameters are often irretrievable, such as the temperature of the body at the time of death (perimortem), or the ambient temperature prior to discovery of the body. This lack of information increases the uncertainty interval associated with the reconstructed PMI [30,31], thereby reducing its forensic value. Consequently, ensuring forensic relevance of the reconstructed PMI in such cases necessitates narrowing of this uncertainty interval. This, in turn, can be achieved by determining which set of thermodynamic input parameters yields *simulated* body temperatures most closely resembling the *measured* body temperatures. To this end, parameter optimization routines can be used, which *minimize differences* (in the form of a cost function) between the simulated and the measured thermometric data, by varying the thermodynamic input parameters. Conventional parameter optimization strategies often rely on the frequent evaluation of this cost function (e.g. to estimate its gradient), and hence on a large number of simulations. As such simulations are generally time-consuming, they preclude rapid evaluation of the cost function. This, in turn, renders conventional parameter optimization strategies (in conjunction with postmortem body cooling simulations) unsuitable for forensic practice. To overcome this challenge, we use surrogate model-based parameter optimization, which straddles the divide between effective optimization and computational cost [32–34]. In this type of optimization, a *surrogate (model) of the cost function* (SCF) is constructed (and continuously updated) by *interpolating* between a limited number of cost function values (thereby reducing the required number of time-consuming cost function evaluations). The evaluation of this SCF is much faster and therefore allows rapid and comprehensive parameter variation. In this work, we combine surrogate model-based parameter optimization, non-invasive three-dimensional thermometry and numerical simulations of the postmortem temperature of recently deceased human bodies to reconstruct their PMI. Using this approach, we demonstrate, for the first time, the feasibility of accurate thermometric PMI reconstruction when the ambient and the perimortem body temperature are unknown.

## 2. Results

### 2.1. Thermometrically estimating the time since death when the ambient and the perimortem body temperature are unknown

Our method exploits the cooling kinetics of a recently deceased human body to estimate (i) the time since death, (ii) the ambient temperature prior to discovery of the body and (iii) the perimortem body temperature. Here, this information is determined by iteratively comparing *measurements* and *simulations* (see also *Methods*) of the skin temperature as a function of the PMI (i.e. measured and simulated skin temperature *curves*). For the simulations, we use a three-dimensional thermodynamic finite-difference (TFD) algorithm [29] (which computes the *thermal energy* exchanged between the body and its surroundings), allowing calculation of the postmortem temperature (at any body location) as a function of the PMI. This algorithm requires (i) a three-dimensional model of the body, (ii) the thermal properties of all involved materials (e.g. thermal conductivity, specific heat capacity, etc.) and (iii) the ambient and the perimortem body temperature. In this study, the necessary three-dimensional models were generated using Structure-from-Motion (SfM) [35,36], while all thermal properties were derived from literature [37–40]. Finally, in order to determine the unknown ambient and perimortem body temperature, we employ a surrogate model-based parameter optimization scheme. In this two-tiered approach, both unknown parameters are varied (within finite bounds, see also *Methods*) until the difference between the measured and the simulated postmortem skin temperature curve is minimal.

**Figure 1.**
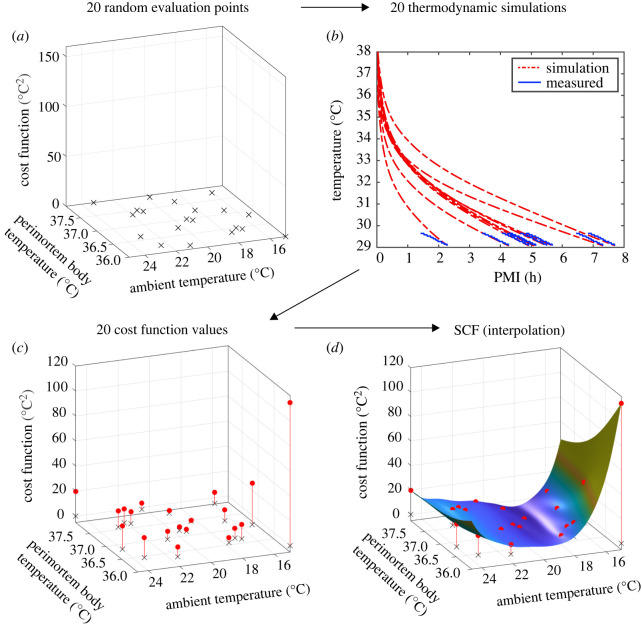
Initial construction of the SCF. (*a*) Twenty perimortem and ambient temperature combinations (referred to as *evaluation* points and shown as black crosses) are chosen randomly. (*b*) Using these 20 parameter combinations, 20 skin cooling curves are simulated (red dash-dotted lines). Next, the sum of the squared differences between these simulated and the measured temperatures (blue solid lines) are computed, yielding (*c*) 20 cost function values (red circles). Finally, using these 20 cost function values, the SCF is constructed (*d*) by means of interpolation (coloured surface).

**Figure 2.**
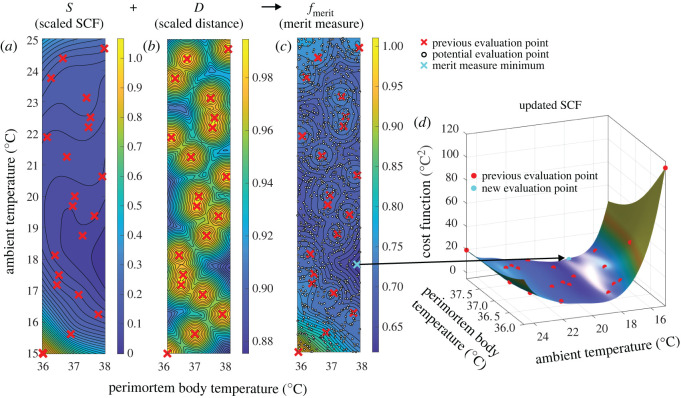
Additional evaluation point selection. (*a*) Scaled SCF *S* as an approximation of the goodness of fit between the simulation and the measurement (for every *potential* evaluation point). Previous evaluation points are shown as red crosses. (*b*) Scaled distance *D* as an inversely proportional measure of the distance between a *potential* evaluation point and *any previous* evaluation point. Previous evaluation points are shown as red crosses. (*c*) Weighted sum of *S* and *D* constituting the merit measure (computed as w⋅S+(1−w)⋅D, and in this case w = 0.3). Previous evaluation points are shown as red crosses. Potential evaluation points are shown as small white circles. The potential evaluation point generating the smallest merit measure value (cyan cross) is chosen as an additional evaluation point. (*d*) SCF, which has been updated using the additional cost function value from the additional evaluation point (cyan circle). Previous evaluation points are shown as red circles.

**Figure 3.**
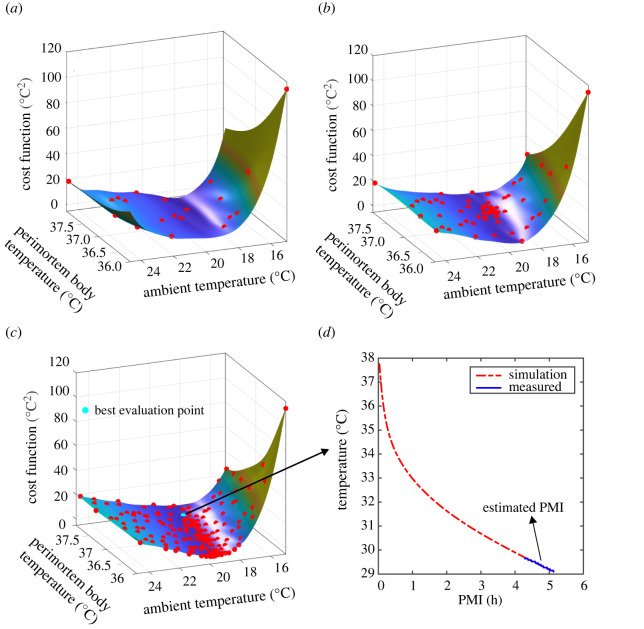
Iterative improvement of the SCF (search for best fit) and PMI estimation. (*a*) SCF (coloured surface) based on the cost function values of 20 evaluation points (shown as red circles). (*b*) SCF based on the cost function values of 80 evaluation points. (*c*) Final SCF based on the cost function values of 200 evaluation points. Among these, the perimortem and ambient temperature combination with the smallest cost function value (cyan circle) generates the simulation which best resembles the measured data (*d*). This simulation then yields the estimated PMI.

The first tier of this approach comprises the construction and continuous improvement of the SCF, which is shown schematically in figure 1. As a first step, 20 perimortem and ambient temperature combinations (referred to as *evaluation* points) are chosen randomly (figure 1*a*) and used to generate 20 (body location-specific) simulated skin cooling curves (figure 1*b*). Comparing these with the corresponding measured skin cooling curves (figure 1*b*) then yields a cost function value (sum of squared differences) for *each* evaluation point (figure 1*c*). Next, using these 20 cost function values, the initial SCF is constructed by means of interpolation (employing a radial basis function interpolator [34,41], figure 1*d*). Following this initial construction, the SCF is continuously updated by incorporating additional cost function values from *new* evaluation points. Ideally, these new evaluation points should both (i) minimize the difference between the simulated and measured skin temperature curve and (ii) evaluate previously untested ambient and perimortem body temperature combinations. These two objectives are balanced in the second tier of our approach, by means of a merit measure (figure 2 and *Methods*). Using this measure, *thousands* of *potential* evaluation points (within the finite bounds) are assessed in terms of (i) their SCF value and (ii) their distance to previous evaluation points. To this end, scaled (i.e. normalized) versions of the SCF (figure 2*a*) and of a distance measure, which is minimal at potential evaluation points far away from previous evaluation points (figure 2*b*), are combined as a *weighted* average to yield the merit measure (figure 2*c*). The choice of weight therefore determines the relative prioritization of the two objectives by the merit measure. Finally, the potential evaluation point associated with the *smallest* merit measure is then chosen as the next evaluation point (figure 2*c*). The corresponding ambient and perimortem body temperature combination is then used to simulate a skin cooling curve, which, in conjunction with the associated measured curve, yields a cost function value (calculated as the sum of the squared differences between the two curves). This additional cost function value is then used to update the SCF by incorporating it into the interpolation (figure 2*d*), which, in turn, improves its approximation of the real cost function. We terminate the search for the unknown ambient and perimortem body temperature after a total of 200 cost function evaluations (i.e. after simulating 200 skin cooling curves based on 200 ambient and perimortem body temperature combinations) and many thousands of merit measure evaluations (figure 3). In this way, the simulated skin temperature curve which best describes the measured curve yields both the reconstructed PMI as well as the previously irretrievable ambient and perimortem body temperature. Crucially, by using the SCF, this two-tiered approach achieves a 15- to 20-fold decrease in computation time as it reduces the required number of time-consuming simulations.

### 2.2. Postmortem interval reconstruction accuracy

**Figure 4.**
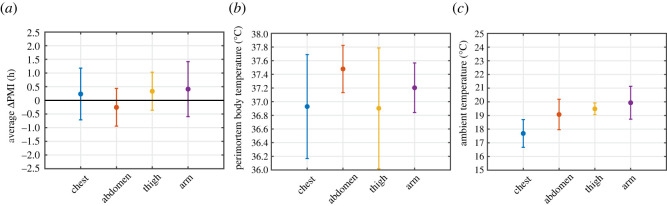
Accuracy of thermometric PMI reconstruction using a surrogate model-based optimization scheme. All averages and s.d. shown in this figure are based on data from four recently deceased human bodies cooling at room temperature. (*a*) Average (±s.d.) of the measurement location-specific PMI reconstruction error (ΔPMI). Combining all errors, we found an average (±s.d.) of 0.18 h ± 0.77 h (11 min ± 46 min). PMIs were reconstructed using measured skin temperatures, in conjunction with a TFD model and a surrogate model-based optimization approach, where the perimortem body and ambient temperature were varied to find the best fit. (*b*) Average (±s.d.) of the measurement location-specific estimated perimortem body and ambient temperatures (*c*), which generated the best fits. We found an average (±s.d.) of 37.13°C ± 0.59°C for all estimated perimortem body temperatures and an average (±s.d.) of 19.04°C ± 1.22°C for all estimated ambient temperatures (which is in agreement with the average measured ambient temperatures of 20.05°C ± 0.47°C).

Figure 4*a* shows the average reconstruction error *Δ*PMI (i.e. the difference between the reconstructed and the true PMI) achieved by the proposed method for each measurement location (chest, abdomen, thigh and arm) across four bodies. The largest measurement location-specific average error was found for the arm with a value of 0.41 h ± 1.11 h, which improves on the findings of a previous study [29]. Individual errors of all measurement locations range from −1.05 h to 1.30 h, with an overall average value of 0.18 h (11 min) and an s.d. of 0.77 h (46 min). These results represent a notable improvement over the accuracy of the standard method, which at best achieves a reconstruction uncertainty of ±2.8 h (and cannot be used if the perimortem body temperature and the ambient temperature are unknown).

### 2.3. Estimated perimortem body temperature and ambient temperature

Figure 4*b* shows the average estimated perimortem body temperature per measurement location. For measurements conducted at the abdomen, we found a slightly elevated average estimated perimortem body temperature, namely 37.48°C ± 0.35°C. These results are in agreement with another study [42], which found an elevated core temperature of 37.6°C in humans shortly before death (in a hospital setting). The average of all estimated perimortem body temperatures was found to be 37.13°C ± 0.59°C, which agrees well with the physiological range of normal body temperatures (normothermia) [43].

The average estimated ambient temperature per measurement location is shown in figure 4*c*. For all measurement locations (except the chest), we found average estimated ambient temperatures between 19°C and 20°C. The chest exhibits a slightly lower average of 17.69°C ± 1.01°C, indicating that simulated temperatures generally exceed measured temperatures for this location, which is in agreement with previous results [29]. This overestimation is likely a result of the simplified body anatomy in our model, which excludes body cavities. As a result, a lower model ambient temperature will minimize the difference between measured and simulated temperatures for this measurement location. Nevertheless, the average of all estimated ambient temperatures was found to be 19.04°C ± 1.22°C, which agrees well with the measured ambient temperatures of 20.05°C ± 0.47°C.

## 3. Discussion

In this study, we developed a powerful analysis approach, which enables, for the first time, non-invasive thermometric PMI reconstruction when the ambient temperature prior to discovery of the body and the perimortem body temperature are unknown. The accuracy of this strategy was evaluated by reconstructing the known PMIs of four recently deceased human bodies (stored at room temperature) and determining the reconstruction error. We found an average error of 0.18 h ± 0.77 h (11 min ± 46 min), which constitutes the lowest PMI reconstruction errors to date. Moreover, we found that the average estimated ambient temperature (19.04°C ± 1.22°C) agreed well with the average measured ambient temperature (20.05°C ± 0.47°C), while the average perimortem body temperature (37.13°C ± 0.59°C) fell within reasonable ranges for normothermia. The above results demonstrate that numerical thermodynamic modelling in conjunction with surrogate model-based parameter optimization can enable accurate and non-invasive PMI determination when the ambient and perimortem body temperature are unknown. Nonetheless, in this study, only one set of ambient conditions was tested (morgue setting). Consequently, future research should establish the accuracy of the technique for other ambient conditions. Similarly, given our limited number of samples, additional comparative validation studies should be carried out in the future. We note that this approach could also be used for other unknown thermodynamic input parameters (e.g. thermal properties). Here, we focused on the ambient and perimortem body temperature, as these are the two parameters least likely to be retrievable in forensic practice. To facilitate the use of our approach by forensic practitioners without knowledge of the underlying programming language, we deploy the software with a graphic user interface. Using this software package, we previously carried out a field validation study at real crime scenes [29], which showed that the use of the software interface by forensic practitioners is easily integrated within existing workflows and requires minimal instruction. We are currently pursuing a fully integrated implementation of our approach, by developing an application for mobile devices (such as tablets) with integrated three-dimensional sensors. This would reduce the required hardware (eliminating the need for a photo camera and laptop) and further simplify and facilitate the use of this approach in forensic practice. In conclusion, next to outperforming the achievable accuracies of the standard method (±2.8 h), our approach may also extend the applicability of thermometric PMI reconstruction to cases *precluding* the use of the standard method. Therefore, we believe that numerical thermodynamic modelling in conjunction with surrogate model-based parameter optimization is a promising strategy ideally suited for future use in forensic practice.

## 4. Material and methods

### 4.1. Non-invasive three-dimensional thermometry of recently deceased human bodies

In this study, we used a photogrammetric (three-dimensional imaging) approach to obtain co-registered anthropometric (shape and posture) and thermometric (skin temperature) data of deceased human bodies. SfM [35,36] was employed to create three-dimensional models of the bodies. To this end, we acquired partly overlapping RGB images of the bodies from different viewing angles, using a standard DSLR camera (Nikon D5300). Scaled three-dimensional models of the bodies were then generated using photogrammetry software (Agisoft PhotoScan Professional 1.2, Agisoft LLC, St. Petersburg, Russia), which geometrically reconstructs points on the body surface, visible from at least two different viewing angles, by means of triangulation. Co-registration of the measured body geometry and skin temperatures at various locations on the body was achieved as follows: coded imaging targets (markers) were attached to four temperature loggers (Thermochron iButtons, Maxim Integrated, San Jose, CA, USA), which were placed at the chest, the abdomen, the thigh and the upper arm, respectively. The locations of these four marked loggers on the body surface were then detected automatically by the photogrammetry software. A fifth temperature logger was used to measure the ambient temperature. For more details on the instrumentation and the measurement protocol please refer to [29].

Recently deceased human bodies were available through the body donation program (BDP) of the Department of Medical Biology, Section Clinical Anatomy and Embryology, of the Amsterdam University Medical Centers (UMC), location Academic Medical Center (AMC), in The Netherlands. The donation of these bodies to science occurred in accordance with Dutch legislation and the regulations of the medical ethical committee of the Amsterdam UMC, location AMC. Written consent (as an extension of an existing codicil) was obtained from all donors, in which they agreed to the use of their bodies and information in taphonomic studies and the publication thereof. Data were collected of four bodies (two male and two female). The subjects' ages, weights and heights ranged from 76 years to 78 years, from 44 kg to 53.5 kg, and from 152 cm to 168 cm, respectively. Upon arrival at the morgue, all bodies were kept at room temperature for the entire duration of the skin temperature measurements. Available PMIs and measurement durations were dictated by the time of arrival of the body at the morgue and ranged from 2.8 h to 7.4 h postmortem, and from 41 min to 290 min, respectively.

### 4.2. Numerical simulations of the postmortem body temperature for thermometric postmortem interval reconstruction

Using the anthropometric data measured by means of photogrammetry, body-specific postmortem body temperatures can be simulated as a function of the PMI [26,29]: in order to generate *spatially resolved* simulations of the body temperature as a function of the PMI, we exploit a three-dimensional finite-difference algorithm in conjunction with an explicit time-stepping scheme. In this TFD model, a three-dimensional cubic grid is rendered to represent the body and its environment. Thermal properties (e.g. thermal conductivity, specific heat capacity, etc*.*) are then assigned to all cubes in the three-dimensional grid, depending on which material they represent (e.g. adipose or non-adipose human tissue, clothing, air, substrate, etc*.*). To this end, the photogrammetrically determined three-dimensional body surface (point cloud) is placed within the three-dimensional grid and thereby transformed into a three-dimensional cubic (volumetric) representation of the body (cube size 1 cm^3^). Importantly, this volumetric representation thus inherently includes both the shape and the posture of the body. Next, every cube is also assigned an initial temperature, e.g. 37°C for the body and 20°C for the environment. These two parameters represent the perimortem body temperature and the ambient temperature, respectively. Note that, using this approach, we can easily account for changes in the ambient temperature by simply adjusting the current temperatures of the relevant cubes. This includes variations *prior* to the discovery of the body (both gradual and sudden). To this end, these variations in ambient temperature can be parametrized using e.g. a periodic/cyclic function or a Heaviside step function, respectively. Of course, this comes at the cost of additional unknown parameter(s) in the optimization routine. Furthermore, our approach makes no explicit or implicit assumptions regarding the absolute value of the ambient temperature and can therefore also be used in cases where the ambient temperature exceeds the body temperature. However, a conceivable limitation in some of these environments may be increased humidity levels, which we currently do not model explicitly in our approach. Next, using our finite-difference algorithm, we calculate the amount of *thermal energy* exchanged between neighbouring cubes for consecutive time steps, which, in turn, yields the *temperature* of every cube for every point in time (i.e. spatially resolved body temperatures as a function of the PMI). By converting the three-dimensional coordinates of the four automatically detected temperature loggers to a location within the three-dimensional cubic grid, we achieve *co-registration of the measured and simulated skin temperatures*. This co-registration step allows highly localized comparison of the measured skin temperatures with their simulated counterparts (i.e. from the corresponding position in the cubic grid). This approach allows PMI reconstruction with average errors not exceeding (and mostly substantially outperforming) the uncertainty of the current standard method (±2.8 h) for PMIs between 2 h and 35 h and for a large range of differences between the measured body and the ambient temperature (1°C to 26°C) [29].

### 4.3. Surrogate model-based parameter optimization

While TFD-based PMI estimation, in principle, only requires a single measured temperature, robust parameter optimization necessitates several, sequentially measured temperatures (i.e. a temperature *curve*). Consequently, in this study, where we optimize two thermodynamic input parameters (the ambient and the perimortem body temperature), we search for the simulated skin temperature curve which best resembles the corresponding (i.e. spatially co-registered) measured skin temperature curve. To this end, we use a surrogate model-based optimization approach (all data analysis was carried out using custom-written code, MATLAB 2020a, The Mathworks Inc., Natick, MA, USA). This type of optimization approach necessitates finite bounds for the varied parameters; here, we chose bounds of 36°C and 38°C, and of 15°C and 25°C, for the perimortem body and ambient temperature, respectively. Following its initial construction, the SCF is continuously updated by incorporating additional cost function values from new evaluation points. Potential candidates for these new evaluation points are assessed by means of a merit measure. The latter returns for each potential evaluation point, a *weighted* average of (i) its *minimization* of the SCF and (ii) its *maximization* of the distance to previous evaluation points. For objective (i) we employ a scaled version of the SCF, which is minimal if the potential evaluation point minimizes the SCF. Here, we use $S(x)=(s(x)-s_{\min})/(s_{\max}-s_{\min})$ where *s*(*x*), *s*_min_ and *s*_max_ denote the SCF value at a potential evaluation point *x*, the minimum and the maximum SCF value across the potential evaluation points, respectively. Therefore, *S*(*x*) ≥ 0 and *S*(*x*) = 0 at potential evaluation points *x* that have minimal SCF value (figure 3*a*). Similarly, for objective (ii), we use a scaled distance measure, which is minimal if the potential evaluation point is far away from previous evaluation points. Here, we use $D(x)=(d_{\max}-d(x))/(d_{\max}-d_{\min})$, where *d*_max_ and *d*_min_ refer to the maximum and minimum distance between all potential evaluation points and all previously evaluated points, and *d*(*x*) refers to the minimum distance of the potential evaluation point *x* to any previously evaluated point. Consequently, *D*(*x*) ≥ 0 and *D*(*x*) = 0 at potential evaluation points *x* that are at maximal distance to any previously evaluated point (figure 3*b*). In order to balance these two objectives, the merit measure takes the form of a weighted sum: $f_{\mathrm{merit}}(x,w)=w\cdot S(x)+(1-w)\cdot D(x)$, where *f*_merit_, *x* and *w*, denote the merit measure, a potential evaluation point and the weight, respectively (figure 3*c*). Here, we cycle through four different weights (i.e. values of *w*), namely 0.3, 0.5, 0.8 and 0.95 as suggested in [33]. The potential evaluation point which best satisfies both objectives, i.e. minimizes the merit measure, is then chosen as an additional evaluation point. Using this additional perimortem and ambient temperature combination, the cost function is evaluated by simulating a cooling curve and calculating the sum of squared differences between the simulated and its co-registered measured curve. This additional cost function value is then incorporated into the interpolation, updating the SCF and improving its approximation of the real cost function (figure 3*d*). If at the end of our search, the cost function values describe a non-convex surface, we regard the parameter search as unsuccessful, as the existence of a minimum cannot be guaranteed. Together, these measures ensure effective optimization (fitting of simulation and measurement) and comprehensive exploration of the parameter space (testing of various perimortem body and ambient temperature combinations). Finally, the skin cooling simulation associated with the lowest cost function value is used to estimate the PMI (figure 4).

## Data Availability

The authors declare that all data supporting the findings of this study are present within the paper. The MALTAB codes and experimental data can be accessed at https://doi.org/10.5281/zenodo.5958450 [45].
